# *In Silico* Docking, Molecular Dynamics and Binding Energy Insights into the Bolinaquinone-Clathrin Terminal Domain Binding Site

**DOI:** 10.3390/molecules19056609

**Published:** 2014-05-22

**Authors:** Mohammed K. Abdel-Hamid, Adam McCluskey

**Affiliations:** Chemistry, Centre for Chemical Biology, The University of Newcastle, University Drive Callaghan, NSW 2308 Australia

**Keywords:** bolinaquinone, clathrin terminal domain, flexible docking, linear interaction energy

## Abstract

Clathrin-mediated endocytosis (CME) is a process that regulates selective internalization of important cellular cargo using clathrin-coated vesicles. Perturbation of this process has been linked to many diseases including cancer and neurodegenerative conditions. Chemical proteomics identified the marine metabolite, 2-hydroxy-5-methoxy-3-(((1*S*,4a*S*,8a*S*)-1,4a,5-trimethyl-1,2,3,4,4a,7,8,8a-octahydronaphthalen-2-yl)methyl)cyclohexa-2,5-diene-1,4-dione (bolinaquinone) as a clathrin inhibitor. While being an attractive medicinal chemistry target, the lack of data about bolinaquinone’s mode of binding to the clathrin enzyme represents a major limitation for its structural optimization. We have used a molecular modeling approach to rationalize the observed activity of bolinaquinone and to predict its mode of binding with the clathrin terminal domain (CTD). The applied protocol started by global rigid-protein docking followed by flexible docking, molecular dynamics and linear interaction energy calculations. The results revealed the potential of bolinaquinone to interact with various pockets within the CTD, including the clathrin-box binding site. The results also highlight the importance of electrostatic contacts over van der Waals interactions for proper binding between bolinaquinone and its possible binding sites. This study provides a novel model that has the potential to allow rapid elaboration of bolinaquinone analogues as a new class of clathrin inhibitors.

## 1. Introduction

The plasma membrane is one of the defining characteristics of all eukaryotic cells. It serves as the boundary between the cellular components and the extracellular environment and facilitates control of cellular material ingress and egress for cells. A plasma membrane comprises a wide array of transmembrane proteins necessary for a range of biochemical processes such as cellular and protein recognition, adhesion, nutrient uptake and signalling [1]. Clathrin-mediated endocytosis (CME) is one of the major processes that regulate selective internalization of important membrane lipids, membrane-bound proteins, hormones and other crucial cellular cargo. These cargo include ion channels, synaptic vesicle and nutrient and growth factor receptors. In addition, CME was reported to have a role in pathogens’ (bacteria and viruses) access to the interior of the cell [2,3]. Cells are also capable of internalizing cargo by phagocytosis, macropinocytosis, caveolin-dependent, and clathrin- and caveolin-independent pathways [4].

CME is the specific process of material uptake using clathrin-coated vesicles (CCV) which arise from the assembly of clathrin-coated pits (CCPs). CCV and CCP formation requires the careful orchestration and interaction of at least 30 proteins with roles at a number of the five different stages of CCP formation: (i) initiation; (ii) cargo selection; (iii) clathrin coat assembly; (iv) scission; and (v) clathrin uncoating, each requiring the synchronization of a wide range of protein-protein interactions to ensure successful cargo internalisation. The CCPs capture their molecular cargo as they bud inward (step (iii)) to form coated vesicles followed by scission (step (iv)), and clathrin uncoating to release the cargo material into the cytoplasm [5]. Structurally individual clathrin heavy chains assemble themselves to form a triskelion like structure with the triskelion apex consisting of clathrin heavy chains (CHC). These heavy chains are extended to form the clathrin three-legged structure. The N-terminal domain (TD) which is folded into a seven-bladed β-propeller lies at the distal end of the leg while the C terminus is near the vertex of the triskelion [2].

**Figure 1:**
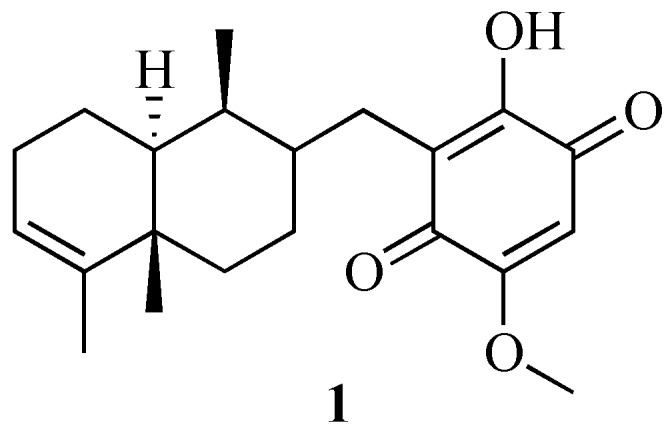
Chemical structure of the marine metabolite bolinaquinone (**1**).

Biochemically a number of human cancers involve clathrin-dependent gene fusion [6,7,8], while defects in CME genes have been linked with the production of epileptic-like seizures in genetic knock-outs [9]. Over the past few years our group has developed a major interest in small modulators of clathrin-mediated endocytosis through the development of the Pitstop*^®^* compound series [2,10]. The marine metabolite, 2-hydroxy-5-methoxy-3-(((1*S*,4a*S*,8a*S*)-1,4a,5-trimethyl-1,2,3,4,4a,7,8,8a-octa-hydronaphthalen-2-yl)methyl)cyclohexa-2,5-diene-1,4-dione (bolinaquinone (**1**), Figure 1) was identified by Casapullo *et al*. as a clathrin inhibitor [11]. While chemical proteomics clearly identified **1** as a clathrin inhibitor, no binding pocket or biological activity (IC_50_) values have yet been reported.

Bolinaquinone (**1**) is a marine sesquiterpenoid derivative first isolated in 1998 from a *Dysidea* sp. [12]. Chemically, **1** comprises a hydroxyquinone C3 head group linked to a trimethyl- octahydronapthalene moiety. While in principle structurally simple, no total synthesis has been reported. This in part is most likely a function of the difficulty in accessing the (4*S*,4a*S*,8a*S*)-3-methylene-4,8,8a-trimethyl-1,2,3,4,4a,5,6,8a-octahydronaphthalene scaffold. Despite this, the presence of comprising two discrete regions suitable for structural modification, and the apparent specific inhibition of clathrin by **1** make it an attractive medicinal chemistry target. The major limitation in developing rapid routes to potent and selective **1**-based clathrin inhibitors is the paucity of mode of action and binding site data. Given our prior efforts in the development of Pitstop*^®^*-1 and Pitstop*^®^*-2 co-crystals with the clathrin terminal domain, we were keen to apply a modeling approach to the rational design of enhanced **1** analogues. Herein we report on our efforts to determine the binding site and potential modes of interaction for **1** in the clathrin terminal domain.

## 2. Results and Discussion

### 2.1. Global Docking of Bolinaquinone into Clathrin TD

**Figure 2:**
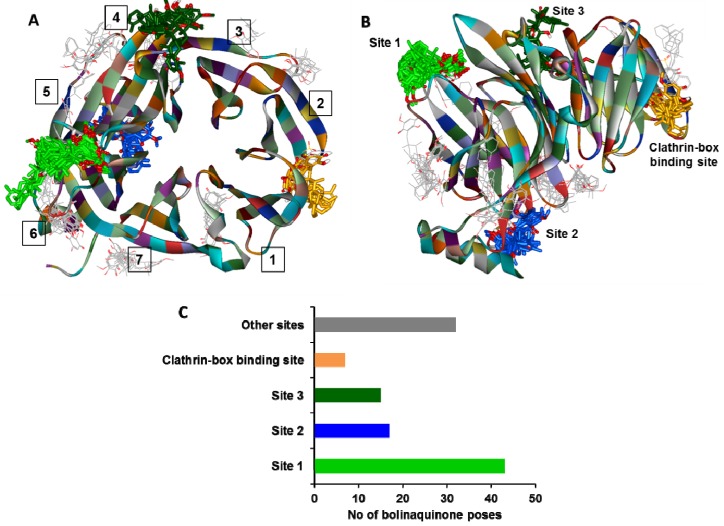
(**A**) Ribbon representation of a top view of the CTD (2XZG). The positioning of each of the seven β-stranded blades is indicated by the numbers 1-7. The predicted binding clusters for the docked **1** poses are rendered in stick representation and are color coded (non-carbon atoms) with 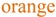 (clathrin-box binding site), 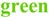 (site 1), 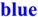 (site 2) and 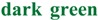 (site 3). (**B**) Same representation as in **A** rotated 90° inward, indicating potential **1** binding sites. (**C**) Bar graph representation of the number of **1** poses in each binding site cluster shown in **A** and **B**. The bar colors correspond to the binding sites and **1**-clusters identified in **A** and **B**.

In order to predict the binding mode of bolinaquinone with the CTD, we first performed a global docking scan with **1** across the entire CTD structure using AutoDock 4.2 [13]. This step identified, and permitted screening of all potential binding pockets for **1**. We had originally hypothesized that **1** would bind at the clathrin-box binding site similarly to what is identified with Pitstop*^®^*-1 and Pitstop*^®^*-2 [2]. Examination of the data from our global docking scan revealed clustering of **1** poses mainly at sites distinct from the clathrin-box binding pocket (Figure 2A,B). These data presumably reflect the structural differences between **1** and either of the Pitstop*^®^* compounds. To validate the global docking approach used with **1**, Piststop*^®^*-1 was docked into the CTD using the same parameters with >47% of the predicted poses clustered co-localizing with the clathrin-box binding site identified by co-crystallization (Supporting Information, Figure S1).

A total of 112 docked poses of **1** at different sites of the CTD were analyzed. The major cluster of **1** poses was found to occupy a potential binding pocket within the CTD propeller blade 5, site 1 (Figure 2C). This site, which was formed by residues Asn175-Val177, Arg221-Lys226 and Gln257-Phe260, was occupied by **1** in 43 poses (38.4% of the total poses). This potential binding pocket was almost at the opposite side from the clathrin-box binding site and was mainly hydrophilic in nature, consistent with the primary binding being driven by the octahydronaphthyl moiety. Further analysis of the docked poses of **1** identified a second potential binding pocket (Figure 2C) between the β-propeller blades 5 and 6 (Figure 2A,B) and was formed by residues Val253-Leu357, Gln203-Glu207 and Gln268-Asp271. The third abundant cluster (Figure 2C) resides within a superficial groove at the interface between β-propeller blades 4 and 5 and was formed by residues Met141-Ser146, Gln182, Tyr184 and Lys189-Ser191. The clathrin-box binding site showed only a low occupancy cluster of seven **1**-poses (6.3% of the overall poses). These binding pose data suggest that **1** occupies a CTD binding pocket distinct from those identified in co-crystallizations studies for Pitstop*^®^*-1 and Pitstop*^®^*-2.

### 2.2. Flexible Docking of Bolinaquinone into Potential Binding Sites at Clathrin TD

A flexible docking approach was used to further validate the potential docking sites identified above and to generate more accurate **1**-CTD complexes as starting structures for MD and binding free energy calculations. The flexible docking experiment was performed using the multistep docking protocol introduced by Koska *et al.* and implemented in the Accelrys Discovery Studio software [13]. Separate docking calculation was performed to place **1** into each of the four potential binding sites. Amino acid residues at a distance of 4 Å from each cluster were treated as flexible during the docking calculations. The obtained docked poses were ranked according to their CDocker energy (kcal·mol^−1^) which was calculated at the final stage of the flexible docking protocol and was used as indication for the binding strength of the ligands (Table 1). Consistent with our initial AutoDock evaluation showing the highest abundant cluster, the potential binding site 1 showed **1** bound with the highest CDocker binding energy of −20.6 kcal·mol^−1^. This provided additional support for site 1 being a valid binding site for **1** within the CTD. The clathrin-box site, contrary to the docking pose occupancy rate identified by AutoDock, showed the second highest CDocker energy of −18.2 kcal·mol^−1^, essentially identical to the −17.8 kcal·mol^−1^ calculated for predicted site 2. Potential binding site 3 returned the lowest CDocker energy of −13.7 kcal·mol^−1^ which is significantly lower than what is predicted for the other binding sites and consistent with the low level of **1** occupancy identified above. The highest ranked poses were extracted from each binding site then were used as starting points for the MD calculations without further modifications.

**Table 1:** Calculated CDocker energies from the final step for the flexible docking protocol of **1** into potential binding sites 1, site 2, site 3 and the clathrin-box site of the CTD.

Potential complex	Flexible residues ^a^	CDocker energy (kcal·mol^−1^)
Site 1	Asn175-Gly179, Arg221-Gln23, Phe252-Phe260	−20.6
Site 2	Val253-Leu357, Gln203-Glu207, Gln268-Asp271	−17.8
Site 3	Met141-Ser146, Gln182-Tyr184, Lys189-Ser191	−13.7
Clathrin-box site	Ile52, Ile62-Ser67, Ile93-Ser97	−18.2

^a^ Residues that were rendered flexible during the docking calculations.

### 2.3. Stability of the Docked **1** Poses

**Figure 3:**
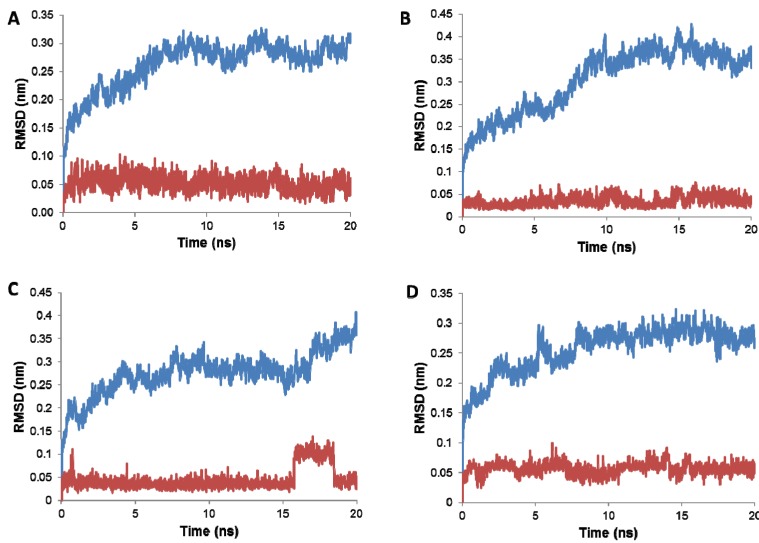
Molecular dynamics trajectory plots correlating RMSD deviation from the initial 1-CTD protein Cα atoms (blue) and **1** heavy atoms (red) coordinates over a simulation time of 20 ns. (**A**) Trajectory plot output for binding site 1; (**B**) Trajectory plot output for binding site 2; (**C**) Trajectory plot output for binding site 3; and (**D**) Trajectory plot output for the clathrin-box binding site. Stable complexes are shown in A, B and D while C shows distortion of the complex structure just after 15 ns.

The stability of the **1**-CTD complex structures obtained from the flexible docking calculations was assessed by probing the stability of the complex via MD simulations. Each complex was subjected to a standard MD protocol with a production phase of 20 ns. The RMSD values for the protein Cα atoms as well as **1** heavy atoms were calculated by aligning the MD production phase trajectories to their initial structures. Examination of the data presented in the RMSD plots show that three of the **1**-CTD complexes corresponding with binding sites 1, 2 and clathrin box were stable during the production phase of the MD simulations (Figure 3). The RMSD values for the three stable complexes (Figure 3A–D) show a convergence of the protein structures after 5-10 ns at average values range between 2.8 to 3.5 Å. In case of **1** docked at site 3, the complex showed significant distortion for both the protein and the ligand after about 15 ns of the production phase (Figure 3C). Inspection of individual frames after 15 ns of the production phase for this complex revealed the ligand, **1**, had been ejected from binding site 3. This finding, combined with the results of both flexible and rigid docking, was consistent with a poor binding affinity for site 3 and thus a low probability that site 3 correlates to the actual **1**-CTD binding domain. Binding site 3 was thus excluded from further studies. The remaining three **1**-CTD complexes (binding sites 1, 2 and the clathrin-box) were deemed stable and were carried forward for binding energy calculations.

### 2.4. Linear Interaction Energy Calculations

The binding free energy for **1** with its potential binding sites in the CTD was calculated using the ligand interaction energy (LIE) methodology. The energy calculations were extracted from the last 10 ns of the MD production phase for each **1**-CTD complex. The binding site free energy of interaction of the **1** with the CTD complexes was decomposed into van der Waals and electrostatic components (Table 2). The calculated binding energy show a significantly higher affinity for **1** bound at binding site 1 compared to other potential sites. Consistent with the CDocker energy, **1** bound to the clathrin-binding pocket represents the second ranked complex in terms of ligand affinity. Examination of the contributions made by the van der Waals and electrostatic components of the ligand interaction energy for each **1**-CTD complex was consistent with the primary mode of interaction being driven by electrostatic interactions. The electrostatic interaction energy was calculated to be 4-6x that of the van der Waals interaction energy (Table 2). This result was contrary to our initial expectation given the lipophilic nature of trimethyloctahydronaphthalene moiety of **1** and highlights the importance of the benzoquinone moiety in binding to all binding sites identified within the CTD.

**Table 2:** LIE binding free energy (kcal·mol^−1^) and its components for **1** at potential binding sites in the CTD.

Binding site	van der Waals contribution	Electrostatic contribution	ΔG_pred_ (kcal·mol^−1^)
Site 1	−21.5 ± 3.8	−145.9 ± 8.3	−5.3 ± 0.6
Site 2	−16.9 ± 3.2	−72.5 ± 4.7	−2.0 ± 0.7
Site 3	NC ^a^	NC	NC
Clathrin-box site	−18.6 ± 2.9	−103.5 ± 4.5	−3.6 ± 0.4

^a^ NC = Not calculated.

Details of key hydrogen bonding interactions between **1** and binding sites within the CTD over the course of the MD simulations are presented in Table 3. From the data it was clear that electrostatic interactions played a significant role in the binding of **1** to the binding sites identified as stable within the CTD. This was most notable for contacts between **1** and the CTD binding site 1; and **1** and the clathrin box binding site with an average number of hydrogen bonds per frame of 2.4 and 2.2 respectively. Both of the quinone carbonyl moieties of **1** can form stable hydrogen bonds with the CTD and during the course of the MD simulation these were present for 68%–96% of the simulation duration. The hydroxyl group was participated as a hydrogen bond donor in 38%–81% of the analyzed trajectories. For potential binding sites 1 and 2, the methoxy moiety was predicted to display minimal interactions in hydrogen bonding (15% of the analyzed trajectories), but participate strongly in hydrogen bonding interactions within the clathrin-box binding site (67% of the analyzed trajectories).

**Table 3:** Summary of the hydrogen bond contacts between **1** and potential binding sites at the CTD over the course of the MD simulations.

Potential binding site	Hydrogen bond average distances (% existence) ^a^			Average number of H-Bonds ^b^
	Carbonyl groups	Hydroxyl group	Methoxy group	
Site 1	2.4 (96%)	1.8 (65%)	2.1 (27%)	2.43
Site 2	2.9 (68%)	2.3 (81%)	3.1 (15%)	1.72
Clathrin-box site	2.1 (91%)	2.6 (38%)	1.9 (67%)	2.24

^a^ % existence of the hydrogen bonds over the analysis phase of simulation (final 10 ns of the production phase); ^b^ Average number of hydrogen bonds per MD trajectory frame.

The cumulative data obtained herein strongly supports binding site 1 of the CTD as the primary binding domain of **1**. Accordingly the key binding interactions associated with the **1**-CTD complex at site 1 were investigated and analyzed from a drug design prospective.

### 2.5. Key Interactions and Insights for Drug Design

**Figure 4:**
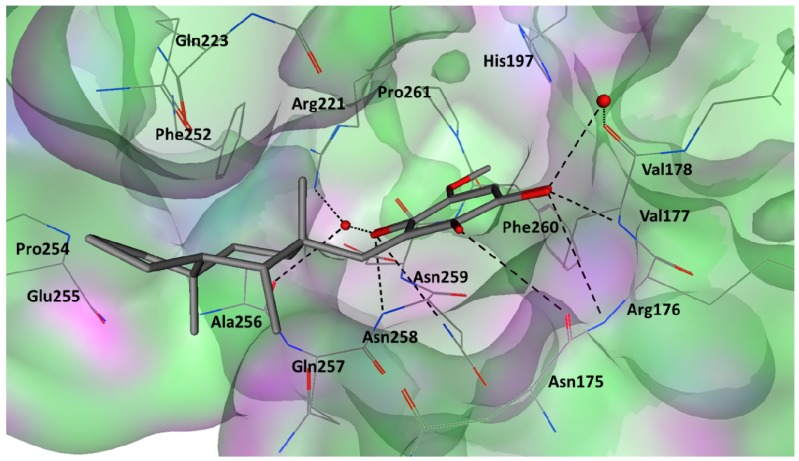
The average energy minimized structure of the molecular dynamics simulated **1** (grey stick representation) bound at the potential binding site 1 of the CTD (only relevant residues are shown as line representation and water molecules are shown as red spheres). Potential hydrogen bonding between **1** and the binding site are shown as black dashed lines.

The **1**-CTD site 1 interactions were investigated in more details based on the average interactions observed during the last 10 ns of the MD production phase. Analysis of the energy minimized average structure re-affirmed the importance of hydrogen bonding at the proposed binding site (Figure 4). Both quinone carbonyl moieties could interact via direct or water-bridged hydrogen bonds from surrounding amino acids including Arg176-Val178, Arg221 and Asn258. With the exception of the bridged hydrogen bonding with Arg221, all observed hydrogen bonds involve the backbone atoms of binding site residues. The hydroxyl moiety of **1** potentially donated a hydrogen bond to the backbone of Asn175. Throughout the MD simulation the methoxy moiety participated in hydrogen bonding (27%, Table 2), but analysis of the energy minimized structure highlighted no hydrogen bonding contacts. However, investigating the individual trajectories suggested the formation of a weak and potentially transient methoxy ‒ water-bridged hydrogen bond with Asn258. The octahydronapthalene moiety displayed weak hydrophobic interactions with Phe252 and Pro254 in agreement with the low calculated van der Waals contribution to the binding energy (Table 2).

**Figure 5:**
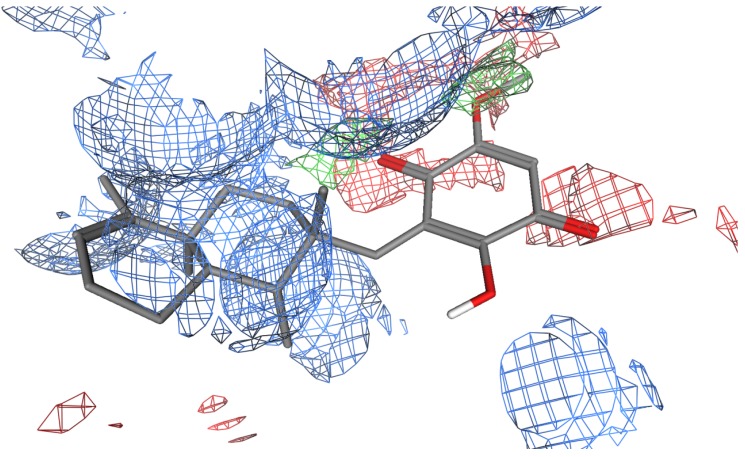
Electrostatics maps (Hydrophobic; green, Hydrogen bond acceptor; red and Hydrogen bond donor; blue) for **1** (stick representation) bound at the proposed CTD binding site.

Examination of the electrostatic potential map that correlated with the binding of **1** at the CTD site 1 was consisted with full alignment of the carbonyl moieties at regions of hydrogen bond acceptor contacts (Figure 5, red mesh) which again emphasizes the importance of this moiety for optimum interaction with the binding site. The hydroxyl moiety is shown in the correct vicinity to participate as a hydrogen bond donor, but not fully aligned for maximal interaction region. This suggested scope for enhanced interaction with key binding site residues through the introduction of a linker between the quinone structure and a terminal hydrogen bond donating moiety. The methoxy group lies in a region comprised of both potential hydrophobic and hydrogen bond acceptor contacts. While seems to be in the right position, the replacement of the methoxy group by a halogen atom or an alkyloxy group is expected to augment the interaction at this position of the binding site. On the other hand, the data shows an unfavorable alignment of the octahydronaphthalene moiety at a region of potential hydrogen bond donor contacts increasing the doubts about its role in binding to the CTD.

## 3. Experimental

### 3.1. Crystal Structure Selection and Preparation

Three different structures of clathrin terminal domain (CTD) bound to the peptide TLPWDLWTT (1UTC), arrestin2s (3GC3) and Pitstop*^®^*-1 (2XZG) were considered as potential starting points for *in silico* analysis of **1**-CTD binding modes. The protein structures were superimposed using the Accelrys Discovery Studio 3.5 software sequence alignment [14] to identify possible backbone discrepancies between structures. Analysis of the RMSD average (2.6 Å based on C_α_ atoms, supplementary data; Figure S2) suggested that no significant differences were present. As 2XZG was reported with a higher sequence completeness and resolution (1.7 Å compared to 2.3 for 1UTC and 2.2 for 3GC3), it was used in subsequent analysis. The bound ligand, water and axillary molecules were omitted and the structure was typed with CHARMm force field. An *in vacuo* energy minimization procedure was performed, after fixing the protein backbone, using steepest descent algorithm for 2000 steps until an energy convergence of ≤0.1 Kcal/mol Å was obtained.

### 3.2. Initial Global Docking

An initial docking step was performed in order to identify potential binding sites for **1** in the CTD. An identical protocol was used to dock Pitstop*^®^*-1 in its known binding pocket as an approach to method validation. Docking studies were conducted with Autodock 4.2 software [13] with a grid box encompassing the complete CTD with a grid spacing of 0.5 Å. For each ligand (Pitstop*^®^*-1 and **1**), 300 docking trials were performed with a maximum of 2,500,000 energy evaluations. On completion, the predicted binding poses were clustered using an RMSD value of 5.0 Å. The most abundant clusters for **1** gave rise to three potential binding pockets in addition to the Pitstop*^®^*-1 binding site (the clathrin-box binding site) (Figure 2).

### 3.3. Flexible Docking

Generally AutoDock software assumes a rigid protein which may affect its accuracy in posing and scoring docked ligands. However how the docking program accounts for protein flexibility is a major factor which influences docking accuracy. Recently scoring and docking accuracy has been was shown to be enhanced when considering, at least, the flexibility of protein side chains in the vicinity of the ligand. Accordingly we applied the Koska *et al.* automated flexible docking protocol within the Accelrys Discovery Studio software [13]. The initial predicted **1**-binding sites were used as the starting structure for re-docking using Koska’s flexible docking approach. Depending on the chosen protocol and for each potential binding site, an initial step of generating side chain conformations for amino acid residues within the binding site was performed using ChiFlex algorithm [15]. Amino acids within 4 Å of each docked cluster obtained from the initial AutoDock predicted binding sites were defined as flexible residues. This step was followed by initial placement of a set of ligand conformations within each of the generated protein side chain conformation using LibDock algorithm [16]. Subsequently, the binding site side chains were refined in the presence of the ligand using ChiRotor algorithm [15]. The final step included simulated annealing (heating to 700 K over 5,000 steps flowed by cooling to 300 K over 10,000 steps) and energy minimization of each ligand pose under CHARMm force field using CDocker protocol [17]. For each binding site, the highest scored complex as defined by the calculated CDocker energy (kcal·mol^−1^) was considered for analysis and as the initial structure for the molecular dynamics step.

### 3.4. Molecular Dynamics Details

Molecular dynamics (MD) simulations were carried out using GROMACS 4.6 software and GROMOS 43a2 [18]. Initial structures were taken as the highest scored pose from each binding site from the flexible docking step (above). The topology and force field parameters were generated for **1** using GlycoBioChem PRODRG2 server [19]. Topologies produced by PRODRG2 server and as with other reports have been used widely without further parameterization in simulating protein-ligand interactions. However, some reports have shown that while the bonded parameters and atom types assigned by PRODRG2 are usually correct, the generated topologies often suffer from inaccuracies in the charges and charge groups that are assigned [20]. Accordingly, partial charges for each docked conformation of **1** were calculated using Gaussian 09 at the B3LYP theory level using the 6-31G** basis set [21]. CTD and the optimized ligand structures were re-joined using the GROMACS “pdb2gmx” procedure at which point the hydrogen atoms were added.

Each CTD-ligand complex was soaked in a cubic box of simple point charge (SPC) water molecules with a margin of at least 10 Å from the protein face. Sodium and chloride counter ions were added to preserve electro-neutrality, preserving a physiological concentration (0.15 M). Each CTD-ligand complex was subjected to a two-phase energy minimization. Phase one employed a 20,000 steps of steepest descent minimization approach and the minimized structure used as the basis of the phase two minimization, a 5,000 step gradient conjugate energy minimization. Subsequently two MD equilibration steps of 500 ps under NVT ensemble using a 2.0 fs integration time step at temperature of 300 K was applied. A second 1 ns NPT equilibration step was executed at 1 bar pressure to equilibrate the size of the system.

In both equilibration steps the C_α_ backbone atoms of the original crystal structure were restrained with all other atoms allowed to move freely. Convergence of the potential energy and system volume was used to ensure adequate solvent relaxation during equilibration. The production phase was conducted using NPT ensemble at 300 K applying the V-rescale temperature coupling algorithm. The pressure of the system was adjusted at 1 atm under isotropic molecule-based scaling using Parrinello-Rahman pressure coupling method. The LINCS algorithm was applied to constrain all bonds while the non-bonded cutoff was set to 10 Å. Long range electrostatics were treated with Particle-Mesh Ewald (PME) algorithm. The time step was set to 2 fs and the energies were collected every 5 ps. The GRID method was used to search and update the neighbor list with a frequency set to 10 steps. The production simulation was run for 20 ns with a total of 10^7^ steps. The produced trajectories were analyzed by plotting the RMSD for each frame against time. The average structure for the trajectories during the stable RMSD region (usually the last 10 ns of the production phase) was calculated for each CTD-ligand complex and was subjected to 5,000 steps of steepest descent energy minimization. The minimized average structure was used for further analysis.

MD simulations of free **1** in water were performed to estimate key parameters for calculating binding affinities under a linear interaction energy (LIE) approach. Standard MD simulations were conducted on each possible confirmation of **1** in a box of SPC water molecules with a minimum distance between the ligand and the boundaries of 20 Å. MD simulations for 20 ns were carried out using similar parameters as described above for receptor-ligand complex in the absence of restraints.

### 3.5. Linear Interaction Energy Calculations

LIE is an end-point free energy method for calculating binding free energy [22]. The CTD-ligand binding free energy was estimated from the changes in the electrostatic and van der Waals interaction energies of the ligand with its surroundings as a result of the transfer of the ligand from aqueous solution to the target binding site. In order to maximise method accuracy, the energetic contributions were calculated based on the averages of the conformational ensembles generated by MD simulations of the complex and the free ligand in solution and scaled individually [21]. In the LIE formula, standard scaling factors can be applied for weighting the energetic components; however this does not lead to optimum energy predictions. It is considered of a good practice to use experimental binding affinity data of a training set of known ligands for fitting the scaling factors [23]. The general formula for calculating protein-ligand binding free energy using the LIE method is:

∆G_binding_ = α < E_vdw,b_ − E_vdw,f_ > + *β* < E_ele,b_ − E_ele,f_ > + *γ*

where E_ele_, E_vdw_ are the electrostatic and van der Waals energy terms respectively, “< >” represent the ensemble average over MD simulations trajectory, “b” represents the bound form of the ligand, “f” represents the free form of the ligand, and α, β and γ are LIE fitting coefficients. The equation coefficients α and β were obtained by fitting experimental and estimated binding energies for a series of ten 1,8-naphthalimides clathrin inhibitors (Pitstop*^®^*-1 analogues; Supporting Information, Table S1) [10]. Accordingly, α and β were assigned values of 0.16 and 0.036 respectively. The value of γ was set to 3.2 in order produce a reasonable value for the estimated binding free energy.

The PME algorithm generates non-decomposable in a pairwise manner energy terms requiring re-calculation of the MD production phase of the above experiments using a plain cut-off algorithm. The obtained output trajectories from PME-based MD run were ported to GROMACS to regenerate the required energy file for LIE calculations using the Reaction-Field-zero algorithm for the treatment of long range electrostatics.

## 4. Conclusions

Our use of a combination of molecular modeling techniques has allowed us to rationalize the reported activity of the natural product bolinaquinone (**1**) against the clathrin terminal domain. Applying a protocol of rigid and flexible docking, MD and linear interaction energy calculations we proposed that **1** can possibly bind to four binding sites at the CTD with the binding pocket at the TD propeller blade 5 being most potential. Analysis of the MD and LIE calculations revealed the importance of the electrostatic interactions in the form of hydrogen bonding with the binding site residues compared to hydrophobic contacts for proper enzyme binding of **1**. This highlighted the importance of the quinone moiety and its hydroxyl and methoxy substituents for proper CTD binding while suggesting a lesser role for the octahydronaphthalene moiety in binding to the CTD. This model represents a valuable tool for the future design and synthesis of simplified bolinaquinone analogues as potential chemical biology probes to dissect clathrin terminal domain function and as potential future therapeutic agents. We, and others have had considerable success in applying such virtual screening approaches in the development of more potent and selective inhibitors against a range of different targets [24,25,26,27,28].

## Supplementary Materials

Supplementary materials can be accessed at: http://www.mdpi.com/1420-3049/19/5/6609/s1.
