# Supplementary material for: In Silico Docking, Molecular Dynamics and Binding Energy Insights into the Bolinaquinone-Clathrin Terminal Domain Binding Site
Source: Molecules. 2014 May 22;19(5):6609–22. doi: 10.3390/molecules19056609 (PMC6270888; doi:10.3390/molecules19056609)
Supplement: Supplementary file 1 [file molecules-19-06609-s001.pdf]

Supplementary Materials

Figure S1. Clustering of pitstop 1docked poses (stick representation) into clathrin TD (3XVG). The major cluster (green) is shown at the clathrin-box binding pocket.

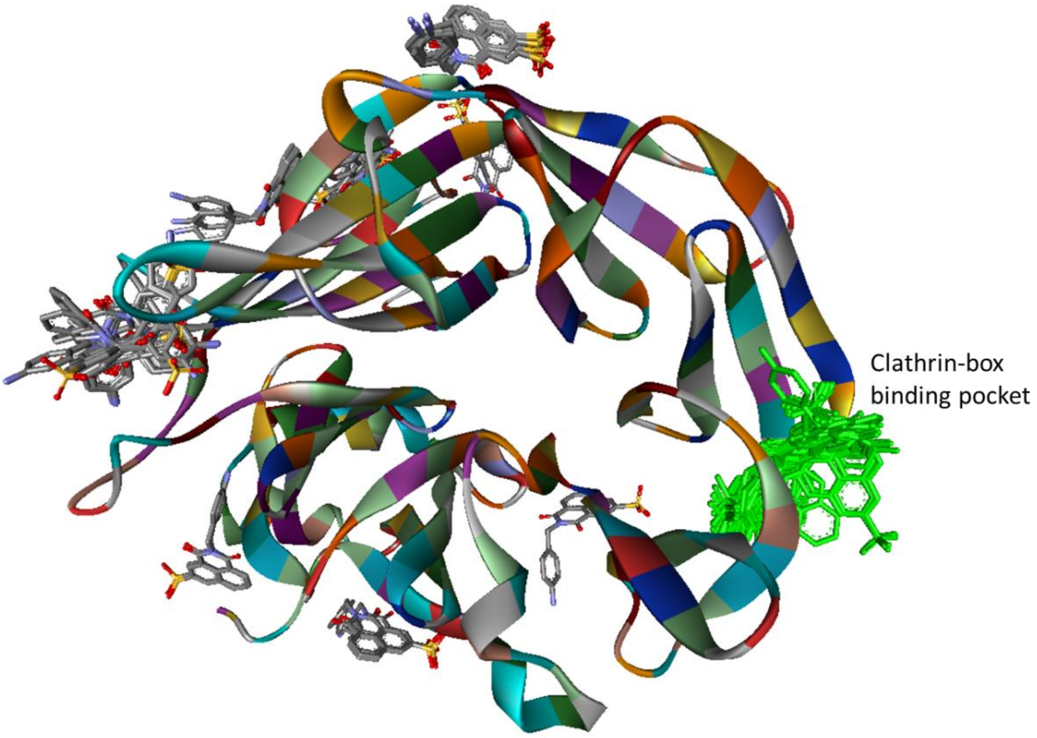

Figure S2. Superimposition of the CTD backbone (ribbon representation) for crystal structures 1UTC (orange), 3GC3 (green) and 2XZG (blue) showing identical orientation for major enzyme loops.

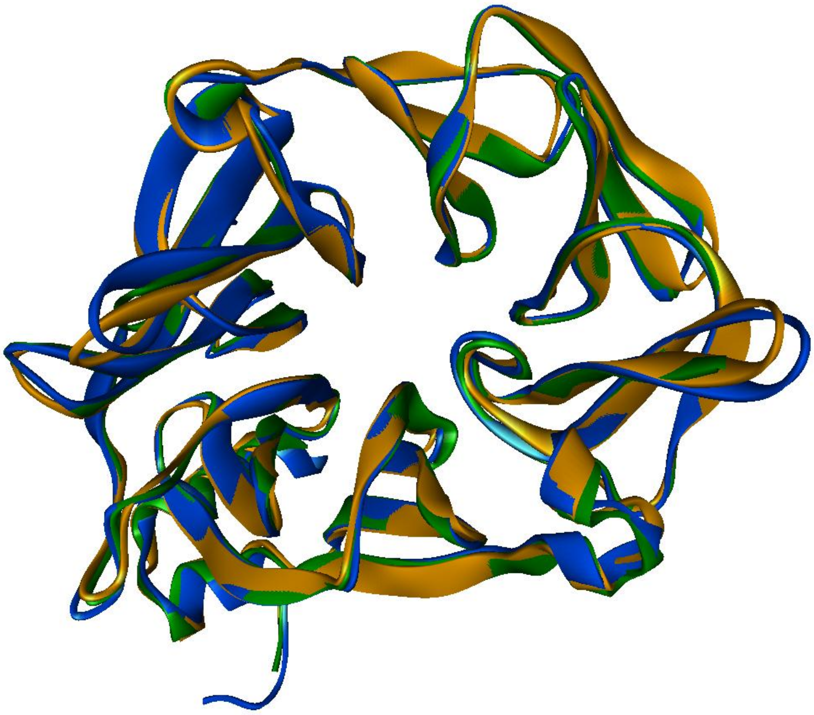

Table S1. Experimental and LIE binding free energy (kcal/mol) for Pitstops at the clathrin TD.

Compound No. [a]	IC_{50} [a]	ΔG_{exp} (kcal/mol)	ΔG_{pred} (kcal/mol)
18	18	−5.60	−5.51
24	10	−6.42	−6.53
25	6.9	−6.94	−7.15
26	22	−5.32	−5.21
27	16	−5.77	−5.54
28	15	−5.86	−6.1
32	12	−6.17	−6.32
33	10	−6.42	−6.45
34	15	−5.86	−5.47

[a] Compound numbers and activity data are according to reference s1.

Reference

S1. MacGregor, K.A.; Robertson, M.J.; Young, K.A.; von Kleist, L.; Stahlschmidt, W.; Whiting, A.; Chau, N.; Robinson, P.J.; Haucke, V.; McCluskey, A. Development of 1,8-naphthalimides as clathrin Inhibitors. *J. Med. Chem.* **2014**, *57*, 131–143.
